# Psychological Sequelae of Dog Bites in Children: A Review

**DOI:** 10.3390/children11101218

**Published:** 2024-10-07

**Authors:** Laura Monti, Georgios D. Kotzalidis, Valentina Arcangeli, Camilla Brozzi, Rossella Iacovino, Cristina Giansanti, Daniela Belella, Elisa Marconi, Silvia Maria Pulitanò, Marianna Mazza, Giuseppe Marano, Giorgio Conti, Delfina Janiri, Gabriele Sani, Daniela Pia Rosaria Chieffo

**Affiliations:** 1 Clinical Psychology Unit, Fondazione Policlinico Universitario Agostino Gemelli IRCCS, 00168 Rome, Italy; laura.monti@policlinicogemelli.it (L.M.); valentina.arcangeli@policlinicogemelli.it (V.A.); brozzicamilla@gmail.com (C.B.); iacovino_rossella@hotmail.it (R.I.); cristinagiansanti@hotmail.com (C.G.); daniela.belella@policlinicogemelli.it (D.B.); elisa.marconi@policlinicogemelli.it (E.M.); silviamaria.pulitano@unicatt.it (S.M.P.); danielapiarosaria.chieffo@unicatt.it (D.P.R.C.); 2 Unit of Psychiatry, Fondazione Policlinico Universitario Agostino Gemelli IRCCS, 00168 Rome, Italy; giorgio.kotzalidis@gmail.com (G.D.K.); mariannamazza@hotmail.com (M.M.); delfina.janiri@unicatt.it (D.J.); gabriele.sani@unicatt.it (G.S.); 3 Department of Neurosciences, Università Cattolica del Sacro Cuore, 00168 Rome, Italy; 4 Department of Basic Biotechnological Sciences, Intensive Care and Perioperative Clinics, Fondazione Policlinico Universitario Agostino Gemelli IRCCS, Largo Agostino Gemelli 8, 00168 Rome, Italy; giorgio.conti@policlinicogemelli.it; 5 Women, Children and Public Health Department, Catholic University of the Sacred Heart, Largo Francesco Vito 1, 00168 Rome, Italy

**Keywords:** dog bites, childhood, psychological sequelae, post-traumatic stress disorder, perceived trauma, prevention

## Abstract

Background/Objectives: Although rare in the Western world, dog bites may be lethal or lead to physically severe outcomes. However, little attention is given to their psychological consequences. We aimed to review their psychological consequences in children 1–14 years of age, focusing on the prevalence and nature of psychological disorders, evaluating the impact on future mental health of children and their families, and assessing the effectiveness of preventive interventions and measures. Methods: On 23 May 2024, we investigated the PubMed, CINAHL, and PsycINFO/PsycARTICLES databases using (“dog bite” OR animal-induced OR animal-caused) AND (psychol* OR mental OR psychiatr* OR anxiety OR anxious OR depress* OR obsess* OR trauma* OR psychosis OR psychotic OR schizophren* OR schizoaffect*) filtered for ages 0–18 years. This resulted in 311 records, of which 50 were eligible. These included original research, case reports, patient surveys, and reviews/meta-analyses. Results: Findings indicate that younger children are particularly vulnerable, often suffering head/neck bites, leading to severe injuries and psychological distress, with post-traumatic stress disorder (PTSD) being a common outcome. Symptoms such as nightmares, flashbacks, anxiety, and social withdrawal were frequently reported. Positive parental support and timely psychological interventions were found to mitigate these effects. Conclusions: Interdisciplinary approaches integrating education, cognitive restructuring, and behaviour modification are needed to effectively prevent and address the psychological impacts of dog bites. Summarising, dog bites in children result in substantial psychological sequelae, necessitating robust prevention and intervention strategies to improve their quality of life and reduce the risk of chronic mental conditions.

## 1. Introduction

Dog bites constitute an often overlooked health problem in Western countries; children represent the most vulnerable population [1,2]. Dog bites are by far the most frequent type of bite suffered by humans, around 80–90% of all bites in the US [3]. The estimation of death and hospitalization rates varies across countries, depending on variations in dog ownership, but also on how data are retrieved. Pooled data from Australia, Canada, the UK, New Zealand and the United States show mean mortality rates of about 0.05/10,000 and hospital admission rates of about 5/100,000 population [4]. Israeli boys had a higher relative risk for being bitten by a dog [2]. Specifically, a considerable amount of emergency department admissions due to dog bites has been reported in South Australia [5]. Dog bites were the fourth most common reason of injury presentations at the emergency department in South Australia hospital [6]. Dog bite admissions in a UK study accounted for almost 70% of all hospital admissions for mammal bites [7].

Smaller children have higher odds for being bitten by dogs and suffer severe injury, because they are more susceptible to dog attacks to the head and neck. Also, poor cognitive development in children may induce them to underestimate the danger and act impulsively, thus increasing the risk of animal attack [8].

Children in the 1–14-year age range are more at risk than other age ranges [2] and account for about two-thirds of all hospitalisations among young people under the age of 18 years. Two age groups of children were the most bitten [9], those under the age of two [10] and those between the ages of nine and twelve [11].

A US study [12] found that 46% of schoolchildren had been bitten by a dog before reaching primary school, leading to aesthetic and psychological consequences that can affect their quality of life (QoL) and that of their families [13,14]. Indeed, bites can result in serious physiological and psychological complications, including post-bite cellulitis, facial disfigurement, neurovascular and musculoskeletal injuries, post-traumatic stress disorder (PTSD), and eventually death [15,16,17]. However, there is dearth of studies evaluating the occurrence of severe mental disorders following canine aggression.

Despite dog bites being a common cause of injury among children, leading to significant physical and psychological consequences, the mental repercussions of these incidents are often underestimated in the scientific literature. This oversight is especially concerning given the high prevalence of psychological trauma, including post-traumatic stress disorder (PTSD), that can result from such experiences. Children, especially younger ones, are particularly vulnerable to this type of trauma due to their limited ability to recognize danger signals and their ongoing emotional development, which makes them more susceptible to long-term consequences.

A review of studies on the psychological impact of dog bites in children is crucial to address a significant gap in the current research. The urgency of conducting such an analysis stems not only from the prevalence of these incidents, but also from the risk of related psychological disorders going undiagnosed and inadequately treated. Untreated traumatic experiences can adversely affect the quality of life of the children involved, with long-term repercussions on their psychological and social development.

Moreover, in a context where interactions between children and pets are increasingly prevalent, especially in Western families, it is imperative to have tools and strategies in place to prevent and manage not only the physical injuries but also the associated psychological trauma. Without sufficient awareness of these dynamics, many children may be left without the necessary support to overcome the experience of being bitten, thereby exposing them to chronic psychological issues.

Dangerous, frightening, and overwhelming experiences threaten survival and lead to adaptive responses that may result in persistent emotional and behavioural changes resembling those of chronic PTSD. According to the DSM-5/DSM-5-TR [18,19], PTSD is a multiplicity of symptoms developing after exposure to traumatic events and persisting more than four weeks. Exposure to traumatic experiences can affect brain function in different ways and have long-term consequences. The severity of the clinical picture may vary, depending on the traumatic exposure experienced. A study of severely dog-bitten children found important changes in their personalities, such as excessive caution and constricted thinking, which lasted 4 years [20,21]. PTSD in children after animal-induced injuries is common but often overlooked by clinical paediatricians. The low level of diagnostic accuracy is partly attributed to clinicians’ limited awareness of the potential development of acute or chronic PTSD reactions after physical trauma. These paediatric patients are at risk of social, learning, and developmental disorders [22]. They may also experience nightmares, flashbacks, social isolation, anxiety, phobia, low self-esteem, or negative body image problems [22]. Although PTSD is the most reported disorder, other disorders, such as depressive and anxiety disorders, have also been diagnosed [23,24].

Intervention methodologies include traditional one-way adult-to-child educational programs, interactive classroom programs, and self-study lessons using computer-animated tasks. Strategies are thus based on concepts of education, cognitive restructuring, and behaviour modification [21]. Software programs such as The Blue Dog (version 9.3) and the BARK (version 4.31.0) (Be Aware, Responsible and Kind) dog bite prevention programs [25] have been designed to educate both children and parents in the interaction with dogs, so to build an adaptive and protective response in the face of possible dangerous scenarios [26,27].

Investigating the psychological consequences in paediatric patients hospitalised for dog bites is crucial to properly assess and manage their hospitalisation. Currently, there is no Italian national surveillance system for dog bites. Most research has focused on small hospital systems, mainly collecting data from emergency departments and trauma centres.

The aim of this narrative review is to examine the psychological consequences of dog bites in children aged less than one year to 14 years of age. To this end, we conducted a literature search with no time or language restrictions in three databases.

## 2. Materials and Methods

On the 23 May 2024, we searched the PubMed, CINAHL and PsycINFO/PsycARTICLES databases using the following strategy: (“dog bite” OR animal-induced OR animal-caused) AND (psychol* OR mental OR psychiatr* OR anxiety OR anxious OR depress* OR obsess* OR trauma* OR psychosis OR psychotic OR schizophren* OR schizoaffect*) filtered for ages 0–18 years. We included original studies, case reports or series, patient surveys and interviews, and reviews and meta-analyses reporting on psychological outcomes. Excluded were articles not reporting on psychological outcomes, such as those focusing on exclusively surgical outcomes, opinion papers such as editorials, hypotheses, comments and letters without data, and other unfocused or unrelated articles. Eligible papers were assessed and introduced in Table 1, which provides their summary. Eligibility was established with Delphi rounds among all authors; complete consensus was required. Delphi rounds are encounters among all participating authors where a leader (LM) provides explanations as to which are the basic concepts of study eligibility and all members decide whether a given study may be included or not. If consensus is not reached, the encounters are repeated until full consensus has been reached. This has been reached after two rounds.

## 3. Results

Our search strategy on the aforementioned date yielded 146 articles on PubMed, 69 on CINAHL and 96 on PsycINFO/PsycARTICLES of the American Psychological Association. Of these, 50 were eligible (Table 1, Table 2 and Table 3). These were 20 surveys, 16 reviews of which 2 with a meta-analysis, 5 were cross-sectional (1 also retrospective), 3 retrospective (1 cross-sectional), 2 were case reports, 1 pilot study and 1 experimental study. The 261 excluded studies were 43 matches between PubMed, CINAHL and PsycINFO, 64 with a primary focus on medical-surgical aspects, 34 related but did not report on psychological outcomes, 53 were surgical reports only, 4 were post-mortem studies, 62 were unrelated to the subject matter and 2 not evidence based.

Taking together the studies here included, we obtained an almost complete picture of the physical and psychological consequences of dog bite lesions in children and of the variables involved in such accidents. Cumulatively, results show that smaller children are most vulnerable, with the head and neck region representing the most common lesion site. The psychological consequences of dog bites in children are well-documented and may include the development of post-traumatic stress disorder (PTSD) symptoms [21,29]. Positive parental support has been found to attenuate these consequences [20]. Therefore, timely psychological interventions are needed focusing on PTSD prevention and addressing long-term psychological consequences [22]. Studies also underscore the importance of knowing the child’s temperament and, most importantly, their impulsivity; prevention should focus on discouraging the child from establishing close contacts with the animal [35]. Furthermore, a better understanding of the dog’s behaviour is crucial in letting the educators to instruct the child on whether to approach the dog or not [10]. Most studies welcome psychological interventions to reduce the risk of dog bites and consider interdisciplinary interventions most suitable for preventing and handling dog bites in children. 

**Table 2:** Summary of eligible studies on the psychological effects of dog bites in chronological order. Included are reviews and reviews/meta-analyses (marked by RMA in the Author(s) year box).

Author(s) Year	Objective	Methods	Results	Conclusions
Voelker, 1997 [12]	To examine the issue of dog bites as a public health concern and identify efforts needed to prevent and manage them	Analysis of dog bite data in the US was conducted, focusing on annual estimates of severe bites and their public health consequences	In the US, 2% of the population is bitten by a dog each year, with approximately 800,000 severe bites requiring medical attention. Children are particularly at risk	The consequences of dog bites can be severe and include disfiguring wounds and, in extreme cases, death. There is a need for commitment to educate dog owners about responsibility and provide information on dog training and socialisation
Sacks et al., 2000 [13]	To summarize 20-year data on dog breeds involved in fatal attacks for assess policy decisions	Data on fatal dog attacks from 1979–1996, along with new data from 1997–1998 were compiled using sources and The Human Society of the United States’ registry databank	1997–1998: 27 reported deaths from dig bite attacks. Over the past two decades, at least 25 breeds have been implicated in 238 human fatalities	Fatal attacks involving pitbulls and Rottweilers stand out. These fatal attacks represent a small fraction of overall dog bite injuries to human. Many alternative measures to breed-specific laws offer potential for preventing dog bites
Calkins et al., 2001 [15]	To analyse children who have experienced life-threatening dog bites, determining the primary aspect of injury	39 children admitted to the trauma service at a regional paediatric trauma centre with diagnosis of dog bite injury > 6 year period (1994–1999). Median age: 5.4 years	71% injuries occurred head/neck region. 23% children sustained life-threatening injuries. 20% arterial occlusion requiring vascular reconstruction. 20% bleeding to death	Significant impact of blunt force injuries, which can lead to severe acute arterial, brain and spinal cord damage. Potential occult blunt arterial or neurological injuries should be ruled-out
Ozanne et al., 2001 [4]	To analyse Australian dog bite injury data and make international comparisons, risk, protective factors related to dog and injured person	Data extracted from routine health records and vital statistics. International comparison data derived from published reports and electronic and bibliographic searches	The Australian dog bite death rate is ↓ than US and Canadian rates. There was a decline for children < 5 year. Children 0–4 ↑ rate of serious injury, particularly facial. Longer hospitalisations for adults	Risk factors: child, male, households with dogs, certain breeds, male dogs, home location and leashed dog. Solutions include promoting responsible ownership, separating children from dogs, high-risk breed avoidance, and neutering
De Bellis et al., 2005 [36]	To provide a comprehensive review of paediatric PTSD. Special emphasis placed on children-specific developmental and neurobiological factors	Literature review of PsycINFO and Medline databases. Each subject heading explored in detail, including trauma history, treatment, epidemiology, clinical presentation, assessment, and neurobiology	Children can experience trauma in various forms, including interpersonal and non-interpersonal trauma. Available effective treatments include trauma-focused CBT	Neurobiological research points to altered stress response and altered activation in brain regions contributing to paediatric PTSD
Abuabara, 2006 [17]	To examine the causes and management of facial wounds caused by dog bites from 1995–2005	Review conducted using the PubMed database, limited to articles published between 1995–2005	Risk factors: school-aged children, males, households with dogs and certain breeds (German Shepherds, Bull Terriers, Rottweilers and Dobermanns)	Facial wounds from dog bites can range from superficial to severe vascular and nerve impairment. Complications may include local infection and sepsis
Jalongo, 2008 [37]	Highlight the importance of integrating safety goals into early childhood education units on pets to prevent dog bites among children	Research from various disciplines to support the effectiveness of education in preventing dog bite injuries to children	400,000/year dog bite accidents in US; young children at highest risk. Education and brief interventions can ↓ these injuries	By incorporating safety goals into pet-themed curriculum, educators can enhance learning and contribute to children’s well-being by promoting safer interaction with dogs
Villalbí et al., 2010 [38]	To analyse population data on dog bite-related hospitalisations after changes in dog ownership laws, including breed-specific regulations	Hospital data 1997–2008 from Catalonia, focusing on dog bite-related hospitalisations post-implementation of dog ownership regulations in 1999–2002	↓ hospitalizations for dog bite injuries following stricter dog ownership regulations in 1999/2002, greatest ↓ in rural areas	After Catalonia enacted new stricter regulations severe dog bite injuries ↓, indicating the potential effectiveness of regulatory measures in ↓ such accidents
Horswell et al., 2011 [39]	To evaluate the epidemiology of dog bites injuries in the Charleston Area Medical Center, Charleston, West Virginia	The study involved 40 children (21 boys, 19 girls) with mean age of 5 years. Injury Severity Scales were used	↑ injuries were caused by familiar dogs in rural areas. Many children suffered severe facial injuries requiring surgery and hospitalisation, with post-surgical complications in some cases	↑ injuries were soft tissue-related; however ↑ severe bites and injuries occurred in pit-bull and Rottweiler attacks. Children < 5 years suffered ↑ injuries requiring medical treatment
Boat et al., 2012 [40]	The study observed 34 paediatric dog bite victims and their families 1 month after receiving care in a paediatric ED: 70% of parents noted new, alarming behaviours in their children post-bite; 85% expressed concerns about their own reactions	Participants were identified using a live computer tracking system and enrolled by a trained clinical research coordinator. Inclusion criteria: age (0 months–16 years) and a presenting complaint of “dog bite”. Parents completed a questionnaire. 4 weeks later, underwent a 30-min phone interview	50 participants enrolled, 34 completed interviews. Most bites occurred outside. 80% unwitnessed. 53% received follow-up care, half in ED. Most suggested extra help times: at ED or doctor’s office, when a child starts healing and after healing with scars and fears. 41% had contact with other service agencies. Parents identified medical, legal, insurance, and animal control systems for assistance	Post-bite, both children and parents showed behavioural alterations. They both need additional help and timely support. Education on dog bite prevention and safety measures will reduce future accidents
Kassam-Adams et al., 2013 [23] RMA	To examine risk factors and preventive strategies for persistent PTS in children following paediatric injury	Meta-Analytic and prospective data were analysed to identify risk factors associated with persistent PTS	⅙ Children and their parents develop persistent PTS symptoms following animal bites. Children’s subjective appraisals of the injury significantly influence PTS onset	Findings underscore the importance of “trauma-informed” paediatric care in addressing acute reactions and preventing persistent PTS in children following paediatric injury due to animal bites
Chiam et al., 2014 [5]	To detail the attributes, contexts, and repercussions of dog bite injuries in children	Retrospective analysis of children who presented to the ED of WCH from 2009–2011	During 2009–2011, 277 children sought treatment at WCH for dog bite injuries. ↑ injury rates observed in children aged 0–4. Head/neck region most affected	≈50% of children required ≥ 1 operation under general anaesthesia and 2 were referred to a psychologist to manage PTSD
Touré et al., 2015 [41]	A 13-year prospective study to identify risk factors for dog bites	Data analysis with the Epi Info Version 6.04dfr software to identify correlations between the studied factors and face dog bites, which are the most detrimental	Dog bites to the face constituted a small percentage of emergency admissions, with children aged 2–5 years. The injuries varied in severity and often involved multiple wounds	The specific risk factors here identified may help implementing concrete primary prevention measures against dog bites
Murray, 2017 [22]	To promote prevention of dog bites and healthy interactions between children and dogs through a literature review	The literature review explored recommendations for the management of dog bite injuries in children	Psychological well-being of children and families affected by dog bite injuries is a main focus. Despite being used routinely, prophylactic antibiotics do not prevent infection, except in hand injuries	The article underscores the importance of implementing clear guidelines for professionals regarding reporting and safeguarding procedures for dog bite injuries in children
Ng et al., 2017 [42]	To analyse treatment for paediatric scalp avulsion injuries from dog bites, with 2 patient examples	2 cases where scalp avulsion was treated with debridement, early wound coverage, and multistage secondary revision using serial tissue expansion and excision	Both patients, with initial loss of 70% and 40% of hair-bearing scalp, achieved satisfactory aesthetic outcomes at 3-year follow-up with no infective complications	Paediatric scalp avulsion injuries from dog bites often preclude replantation
Shen et al., 2017 [43] RMA	To assess the efficacy of cognitive/behavioural interventions in enhancing children’s knowledge and behaviours concerning dog-bite prevention	Children < 18 considered. From 2270 abstracts screened, 123 full texts were reviewed; 12 studies included in qualitative synthesis; 9 in the meta-analysis. Risk of bias and evidence quality were assessed	CBT moderately improved children’s knowledge and promoted correct behaviour with dogs. Video-based approaches were effective for knowledge, while live dog instruction was most beneficial for behaviour. However, quality of evidence was low	CBT offer promise in enhancing children’s knowledge and correctness of behaviour regarding dogs
Jakeman et al., 2020 [7]	Review current evidence-based practices, analyse patterns in biting accidents, and discuss efficacy of prevention strategies, shed light on the physical and psychological complexities of dog bite injuries, describe the overlooked psychological effects on both children and caregivers	The review compiled data from various sources, including statistics of hospital admissions, regional variations in hospitalisation rates for dog bites, demographic trends of victims, and existing research on the incidence and impact of dog bites	Hospitalisation rates for dog bites vary regionally with ↑ rates in more deprived areas, such as Merseyside, England. Children <9-years-old are most affected by dog bites, with ⅔ of admissions among <18-years-old. Children <2-years-old and those aged 9–12 years are the most frequently bitten age groups	This work advocates supporting environmental changes to ↓ the likelihood and severity of dog bites. Moreover, the article emphasises the importance of psychological support for bite victims and their families to address long-term impacts
Lantz, 2020 [44]	To explore long-term psychological effects of accidental trauma on paediatric patients and their implications for nursing care. The focus tends to be on physical recovery, leaving psychological sequelae unaddressed	This article examines studies that address the prevalence of PTSD, depression, and anxiety disorders following traumatic events in children. Additionally, it discussed the role of nurses in providing care for paediatric trauma patients and proposes the use of trauma-informed care and appropriate screening techniques	Despite the well-documented connection between paediatric accidental trauma and long-term psychological effects, screening rates and referrals for psychological services remain consistently low. This lack of intervention leads to long-term negative impacts on children’s physical, emotional, cognitive, and developmental well-being	The specific effects of PTSD in children vs. adults should be further explored. Randomised control trials could help developing prevention and treatment strategies. Nurses may help reducing the impact of trauma on children and prevent mental health issues following accidental trauma

Abbreviations: CBT, cognitive-behavioural therapy; ED, Emergency Department; PTS, post-traumatic stress; PTSD, post-traumatic stress disorder; US, United States of America; WCH, Women’s and Children’s Hospital of South Australia; ↓, decreased, low; ↑, increased, high.

**Table 3:** Summary of eligible studies on the psychological effects of dog bites in chronological order. Included here are surveys.

Author(s) Year	Objective	Methods	Results	Conclusions
Rossman et al., 1997 [20]	To investigate and determine factors predicting the occurrence of PTSD symptoms and the adaptive functioning of children exposed to dog bites	86 parents and their children, aged 4–9 years, were interviewed through PTSD-RI, CDC and CBCL	↑ trauma symptoms with younger age and female sex.	Children exhibit ↑ PTSD symptoms after ≥ 1 bites. Positive parental support correlates with ↓ symptoms and improved adaptation
Thompson, 1997 [6]	To assess the impact of dog bites, determining the incidence and risk factors associated with dog attacks	Queen Elizabeth Hospital, Adelaide, South Australia, 1990–1993. 356 victims of dog attacks who presented to the ED and 3093 respondents to the 1992 South Australian Health Omnibus Survey	6500 people are injured in Adelaide each year as a result of dog attacks: children 0–4 years were attacked and required hospital treatment twice as often as adults 21–59 years, and men > 76 years twice as often as men 36–75 years. Hospital admission rates were ↑ × 5 for the elderly and ↑ × 7 for children ≤12 years compared with people aged 13–59 years	The public health impact of dog attacks is significant, especially concerning young children. Proposed interventions: stricter controls on high-risk breeds, mandatory leash laws and implementing insurance system
Bernardo et al., 2002 [45]	To determine if differences exist in agent, host, and environmental characteristics among younger and older patients treated in a paediatric ED for dog bites	Patients in 1999–2000 were identified through a review of ED records (n = 386) of children suffering dog bites. Records were extracted in a researcher-designed and validated form	Children < 6 years constituted 52.8% (n = 204) of the sample. Younger children were bitten by their family dogs, on the face, in their own homes	Findings from this study could be used to develop age-specific strategies for dog bite prevention
Peters et al., 2004 [16]	2001–2002, children bitten by dogs were consecutively enrolled at the ED of UCH. Inclusion criteria: <16 years; having only minor surgery within 48 h from dog bite	Parents agreed to complete a questionnaire 2–9 months after the accident. DSM-IV PTSD diagnosis. Statistical analyses with non-parametric tests	22/26 children, with a median age of 7.5 years old, met inclusion criteria. 50% boys. 7 months post-accident, 12 PTSD symptoms lasting >1 month	Children experiencing multiple/deep wounds from violent dog attacks more likely to develop PTSD. Some children displayed aggressive play imitating dogs or were reluctant to go out alone. Even minor bites cause emotional distress
Hon et al., 2007 [46]	To analyse the pattern of dog bites presenting at the ED of a university hospital	Patients < 22 years, evaluated at the ED with a discharge diagnosis of animal bites, were identified through a computerised discharge network	144 cases of animal bites, recorded at the ED, were mostly from dog bites (89%). Children <10 years ↑ facial injuries and were often triaged as urgent. Symptoms: pain, bleeding, bruising	Young children are at a ↑ risk of facial injuries. Pain severity and psychological impacts are often overlooked in emergency assessments. Hospitalisation was usually unnecessary
Ji et al., 2010 [21]	To analyse the incidence and predictors of PTSD after dog bite injuries	Data for 358 children injured by animals. PTSD screening and Family Apgar Scale assessment conducted upon ED admission. AcStD diagnosis within the 1st week using the CASQ. 3 months later, PTSD screening using the CAPS-C-A	19 patients developed PTSD. There were no significant gender or age differences in PTSD occurrence. Family Apgar Scale scores did not show a significant association with PTSD. However, there was a significant correlation between AcStD and PTSD symptom severity scores	Severe animal attacks can lead to PTSD in children. Family support does not seem to affect PTSD symptoms in school-aged children after such attacks. Early symptoms of AcStD may predict later development of PTSD
Lakestani et al., 2011 [47]	To develop and conduct an initial assessment of a dog attitude scale for preschool children and adult across European nations	Brief questionnaires to 107 nursery school children (mean age 4.5 years) and 120 university students (mean age 21.3 years) in Milan, Barcelona and Edinburgh	Dog ownership correlated with positive attitudes in both children and adults. No significant differences were found based on country or gender	A single questionnaire can assess young children’s attitudes towards dog, suggesting a uniform approach to dog bite prevention and help education programs
Yeh et al., 2011 [48]	Case-control studies to assess whether individuals with mental disorders are predisposed to dog bites and subsequent post-bite cellulitis	Case-control studies 2000–2007, comparing 4660 patients with dog bites to 18,640 controls without dog bites. Additional comparison between 286 patients with and 4374 patients without post-bite cellulitis	Children, older adults and individuals with ↓ socioeconomic status at ↑ risk of dog bites. Patients with both psychotic and non-psychotic mental disorders had ↑ risks of both dog bites and post-bite cellulitis	Individuals with mental disorders face an ↑ risk of severe dog bites and subsequent post-bite cellulitis
Davis et al., 2012 [35]	This study investigated the effects of children’s temperament on their interaction with dogs and how it can be a risk-taking for the children	88 children 3.5–6 years interacted with a live dog. Dog and child behaviours were assessed through observational coding. 4 child temperament constructs were assessed via the parent-report CBQ	Less shy children took ↑ risks with the dog, even after controlling for child and dog characteristics. No other temperament traits were associated with risk-taking with the dog	Dog bite intervention programs to target children at risk; parent-oriented interventions
Vargo et al., 2012 [49]	To delineate a pressing public health concern in American Samoa, which could potentially affect other regions permitting a substantial population of free-roaming dogs	A dataset (patient from 2004–2010) from the only ED in the Territory was analysed. A survey of 437 adolescents was conducted to document their encounters with dogs attacking them unprovoked during the 2010/2011 school year	Males aged 55–59 years had the highest incidence of dog bites, closely followed by males aged 10–14 years. ≈⅓ of adolescents reported experiencing a dog bite during 2010–2011	Children, adolescents, and elderly people are particularly vulnerable to dog bite injuries. Fear of being bitten by a dog was cited as a factor preventing more physical activity by 10% of males and 16% of females
Eppley & Schleich, 2013 [50]	Describe treatment and outcomes of facial, scalp, and neck dog bites in 107 children over 10 years.	Analysed cases focusing on patient age, relationship with the dog, bite circumstances, and treatment outcomes, including surgeries.	Average age was 5.9 years; 95% knew the dog, 77% of bites were provoked; most wounds closed primarily, 77% opted for scar revision, 39 cases involved lawsuits.	Facial dog bites in children often require multiple scar revisions for best outcomes; families should be informed early.
Morrongiello et al., 2013 [26]	This study focused on how parents supervise and react to their young children about unfamiliar dogs	A randomized controlled trial with pre/post intervention and control groups evaluated whether exposure to The Blue Dog, a program for dog bite prevention and education, improved parent behaviours regarding their children’s interactions with dogs	The study found no significant differences between groups in pre/post-intervention measures, suggesting that The Blue Dog program did not lead to improvements in parents’ behaviours	These findings underscore the significance of addressing parent behaviour, alongside child behaviour, in initiatives aimed at reducing the risk of childhood dog bites. Despite this, The Blue Dog program did not successfully alter parent behaviour
Shen et al., 2013 [8]	To study the contextual antecedents and consequences of paediatric dog bites in rural China	101 caregivers from rural Anhui Province, China, whose children had suffered dog-bite injuries in the past year, participated in a structured interview about the circumstances, antecedents and consequences of their child’s injury	Frequent antecedents were dogs’ initiation of the encounter, children walking to/from school, and dogs unleashed. Frequent consequences were rabies vaccines, restricted activity, and fear of dogs	Results represent a necessary prerequisite for developing empirically supported prevention programs in a vulnerable population
O’Brien et al., 2014 [51]	Identifying patients with dog bites and their impact on health systems	January 2012–June 2013 cohort of an academic tertiary care centre	Pit bull terriers are the dogs most likely to attack children aged 15–18 years, who then require more consultation and more surgery	Children are more susceptible to dog bite injuries to the head and neck
Shen et al., 2014 [52]	Examine the contextual factors leading to paediatric dog bites and their consequences in rural China	Structured interviews of 101 caregivers whose children experienced dog-bite injuries in the past year	Common contextual factors: being outdoors and with no adult supervision. Consequences: rabies vaccines, restricted activity, and fear of dogs	The study provides insights into the prevalent outcomes of paediatric dog bites in rural China
Lakestani and Donaldson, 2015 [27]	To investigate whether preschool children can learn how to interpret dogs’ behaviours, with the purpose of helping avoid dog bites	3–5-years-old children were tested on their ability to answer questions about dogs’ emotional states before and after participating in either an educational intervention about dog behaviour (intervention group) or an activity about wild animals (control group)	Children in the intervention group were significantly better at judging the dogs’ emotional states compared to before. In contrast, the control group’s performance did not differ significantly between the two testing times	Preschool children can be taught how to correctly interpret dogs’ behaviours. This implies that incorporating such training into prevention programs may help reducing dog bite accidents
Ó Súilleabháin, 2015 [53]	To evaluate the effectiveness of current breed-specific legislation in Ireland by analysing all hospital admissions due to dog bites since the implementation of this legislation	Data for statistical analysis were obtained from the National Hospital In-Patient Enquiry Scheme, for dog bites in Ireland from 1998 to 2013	The incidence of hospitalisations ↑ significantly over the years. After dog bites, males had a ↑risk of hospitalisation compared to females. Children <10-years-old were identified as a particularly vulnerable group	The current breed-specific legislation in Ireland is not effective in mitigating dog bites and may be contributing to ↑ hospitalisations
Cohen-Manheim et al., 2018 [2]	To investigate demographic and injury characteristics of dog bites requiring hospitalisation on a national scale	Data on hospitalisations due to dog bite injuries were extracted from the Israeli National Trauma Registry from 2009 to 2016	2009–2016, 986 individuals hospitalised for dog bite injuries in Israel. Children 0–14 years show ↑ hospitalisations compared to other traumas. In 2016, hospitalisation rates are significantly ↑ in boys compared to girls	Prevention programs should target children <15-years-old (particularly boys) who are at a ↑ risk of dog bites
Habarth-Morales et al., 2022 [54]	During the COVID-19 pandemic, reports from various centres suggest an ↑ in dog bite injuries, particularly in paediatric populations	Authors examined hospital’s electronic health record and NEISS for dog bite records from 2015 to 2020 and calculated the annual incidence	Both institutional and national cohorts showed relative ↑ in dog bite injury incidence over the study period. Significant ↑ of 44% and 25% in annual incidence relative to 2019 observed in both cohorts. The ↑ incidence of dog bites is related to ↑ psychosocial stress of the pandemic condition	Findings underscore the ongoing public health significance of dog bites and highlight the need for continued attention and action from public health agencies to address this issue

Abbreviations: AcStD, Acute Stress Disorder; CAPS-C-A, Clinician-Administered PTSD Scale for Children and Adolescents; CASQ, Child Acute Stress Questionnaire; CBCL, Child Behavior Checklist; CBQ, Children’s Behavioral Questionnaire; CDC, Child Dissociative Checklist; ED, Emergency Department; NEISS, National Electronic Injury Surveillance System; PTSD, post-traumatic stress disorder; PTSD-RI, Post-traumatic Stress Disorder Reaction Index; UCH, University Children’s Hospital Brussels; ↓, decreased, low; ↑, increased, high.

## 4. Discussion

In this review we analysed literature focusing on the psychological aspects of dog bites and found that in recent times there has been an increase in the incidence of bites, especially during the pandemic period, following an increase in the distress of the population and in the dogs themselves. Most psychological sequelae regarded post-traumatic stress, and the related symptoms of anxiety and depression, that if they are not managed early in their development, they may evolve in psychiatric disorders. We found that several programs directed at increasing public knowledge about dogs’ behaviour may decrease the incidence of this increasingly worrisome phenomenon.

A critical examination of dog bite injuries in children reveals multifaceted dynamics that necessitate a comprehensive understanding for effective preventive strategies. The literature underscores the significance of behavioural dynamics preceding dog bites, emphasizing the need for education and supervision. The studies by Reisner et al. [10] and Shen et al. [8] highlight the unfamiliarity of children with the biting dog and the prevalence of unsupervised incidents in domestic settings (although one study found familiar dogs to account for 78% of bites [55]). Such insights advocate for educating both children and adults on appropriate behaviour around dogs and the imperative of supervision.

Moreover, demographic disparities in bite severity and outcomes, as noted by Cohen-Manheim et al. [2], underscore the importance of considering environmental and demographic factors in preventive strategies. Higher hospitalisation rates among boys under 15 years of age point to the need for tailored interventions, taking into account demographic variables. The Belgian experience contributes to understanding canine behaviour and public health concerns, informing preventive measures [56].

Furthermore, the psychological impact of dog bite injuries on children demands closer scrutiny. While post-traumatic stress symptoms have been acknowledged, deeper exploration of PTSD, anxiety, and other psychological sequelae, as well as their long-term effects on children’s well-being, is essential [21,29,44]. Identifying modifiable risk factors associated with post-bite psychological issues could inform targeted interventions and support strategies.

The literature review identified several key psychological outcomes in children who have experienced dog bites, with a notable prevalence of post-traumatic stress disorder (PTSD) and long-term psychological distress. PTSD emerged as the most common psychological consequence, particularly in cases where the bite resulted in severe injuries or involved the face and neck. Common symptoms include traumatic flashbacks, recurrent nightmares, generalized anxiety, and hypervigilance, which, if left untreated, can persist for years, significantly impacting the child’s social and emotional development.

Furthermore, anxiety disorders and specific phobias, such as cynophobia (fear of dogs), were frequently observed, often extending to generalized social anxiety and avoidance behaviours, particularly in younger children. Studies also highlighted the long-term emotional consequences of dog bites, including social isolation, emotional withdrawal, and difficulties in trust, which may lead to a reduction in social engagement if adequate psychological support is not provided. Sleep disturbances, including insomnia and nightmares, were also commonly reported, correlating with PTSD symptoms and further exacerbating anxiety and depression.

It is crucial to consider the impact on the family as well, with parents often experiencing anxiety and guilt, which can affect their ability to manage their child’s recovery. Family stress can, in turn, exacerbate the child’s psychological distress, creating a cycle of emotional strain.

The review’s findings underscore the critical need for early intervention to mitigate the psychological impact of dog bites on children. The prevalence of PTSD, anxiety, and emotional withdrawal following such incidents highlights the importance of implementing standardized treatment protocols across healthcare settings. The variability in how these children are diagnosed and treated, particularly in the absence of long-term follow-up, calls for more consistent and evidence-based approaches. Moreover, the behavioural and psychological repercussions of dog bites can extend into adolescence and adulthood, affecting cognitive and social development.

The results also support the necessity for educational strategies aimed at both children and parents to prevent dog bites and promote safe interactions with dogs. Campaigns focused on raising awareness of the risks and appropriate behaviours around dogs, particularly in domestic settings, are essential in reducing the likelihood of such incidents. Additionally, integrated psychological support within pediatric care settings is paramount, providing children with the necessary emotional and psychological resources to recover from the trauma.

Critically evaluating the effectiveness of existing preventive measures and exploring innovative strategies is mandatory. While advocating educational interventions and parental supervision, a nuanced assessment of their efficacy and implementation barriers is warranted. Variations in programme outcomes across settings and populations, as discussed by Lakestani and Donaldson [27] and Shen et al. [43], offer insights into effective prevention approaches. Additionally, exploring innovative strategies like community-based initiatives and policy interventions could enrich the preventive landscape.

Moving to breed-specific legislations (BSL), contentious debates arise regarding their effectiveness in mitigating dog bite injuries. Opinions diverge, with some people advocating their enforcement [22,57] and some others considering them superfluous [53,58,59], while some others being sceptical and finding advantages to be unclear [60]. Most data-based studies suggest ineffectiveness and potential adverse outcomes [53,59]. All stress the need for evidence-based policy-making and understanding socio-cultural influences on dog bite incidents.

Furthermore, while demographic trends and injury severity are well-documented, deeper exploration into underlying mechanisms is lacking. Disadvantaged populations are more prone to be bitten by dogs within the same geographic area [61], hence a critical analysis of systemic factors like access to healthcare and socioeconomic disparities is warranted to address racial/demographic disparities.

Recognizing the pivotal role of education in fostering responsible behaviour around dogs, there is need to integrate comprehensive programmes into primary school curricula. Insights from a prospective survey provide valuable epidemiological data on children who are victims of dog bites and received treatment in emergency departments, further emphasising the importance of acting proactively through educational interventions [62]. One study, in particular, demonstrated the effectiveness of such initiatives in a randomised controlled trial, highlighting the impact of early education on reducing the risk of dog bites in children [63]. Through making available to children basic knowledge about dog behaviour, safety protocols, and appropriate interaction since a young age, it is possible to empower them to make informed choices and mitigate the likelihood of dog bite incidents. Incorporating these programs into primary education is not just beneficial but mandatory in shaping attitudes and behaviours towards dogs, ultimately safeguarding children’s well-being and fostering a culture of responsible pet ownership from the outset.

Pet therapy can represent an important support for children who were bitten by dogs, helping them overcome the emotional and psychological trauma. It can be extremely useful to introduce training courses on pet therapy to train qualified professionals able to offer effective therapeutic interventions. These courses may prepare professionals to adopt pet therapy techniques to promote emotional recovery, to reduce anxiety and improve the overall wellbeing of children. Furthermore, by integrating pet therapy within the preventive strategies, it is possible to improve the understanding and safe interaction with animals, reducing the risk for future accidents.

In evaluating preventive measures, it is crucial to acknowledge limitations in the existing literature. Methodological shortcomings, including reliance on retrospective data and lack of standardized reporting protocols, reduce the strength of findings. However, this review adds to the existing literature a complete view of the psychological problems arising in children and their environment, as well as possible ways to prevent the phenomenon, without focusing on corrective surgical procedures or individual psychiatric disorders, but rather on the psychological consequences of dog bites and their medical-surgical management.

When, despite preventive efforts, a child is bitten by a dog and is brought to the hospital, a multidisciplinary approach may benefit the child and its parents, Such an approach is adopted in our Clinical Psychology unit, where a multidisciplinary team comprising several professional figures, such as psychologists, paediatric surgeons, and ward nurses guarantees an integrated approach to treatment, considering both physical and psychological aspects of the patient’s sufferance. All those specialists (including paediatric surgeons, paediatric intensive care unit personnel, paediatricians at the Paediatric Trauma Centre, where children with life-threatening injuries are referred, as well as the nurses in their respective wards) who deal with children suffering dog bites face major emotional burden while providing their services; to deal with their emotional turmoil, they receive psychological support from psychologists employed at the Clinical Psychology unit. In particular, psychological support interventions enable the team’s psychologists to help patients in working through their traumatic experience, addressing all the multifaceted emotional, cognitive, and behavioural post-traumatic sequelae of dog bites, and to provide support to the entire family. Every psychologist provides a space for listening and an emotional outlet for patient’s needs; within this space, he/she uses psychoeducation and storytelling techniques, thus ensuring better clinician-patient interaction. Strengthening patient resources enables gradual psycho-physical recovery, simultaneously improving QoL and reducing the risk of future long-term mental disorders.

Summarising, a critical perspective on dog bite injuries in children is paramount for identifying gaps, challenging assumptions, and informing evidence-based interventions. By evaluating existing policies, probing underlying mechanisms, and acknowledging research limitations, we can advance our understanding and develop targeted preventive measures to safeguard children’s well-being. In this frame, the role of the clinical psychologist is central in guaranteeing that the bitten child may overcome the psychological consequences of the dog bite.

## 5. Conclusions

A comprehensive, multidisciplinary approach is crucial for managing dog attacks on children, given their complex and severe psychological impact. Younger children are especially vulnerable to developing post-traumatic disorders like acute stress disorder and PTSD after severe attacks. Psychologists play a pivotal role in supporting both children and families through various therapeutic techniques. Parents are essential for providing emotional and practical support during recovery. Targeted educational programs are vital for preventing dog bites and protecting children. Promoting responsible dog ownership and enacting appropriate legislation are key to reducing incidents and safeguarding children’s well-being.

## Figures and Tables

**Table 1:** Summary of eligible studies on the psychological effects of dog bites in chronological order. Included are case reports, experimental studies, pilot, prospective, cross-sectional and retrospective studies.

Author(s) Year	Type	Objective	Methods	Results	Conclusions
Gislason & Call, 1982 [28]	CR.	To describe the effects of dog bite trauma on 3 children	Clinical observation	In the post-traumatic state, ↑ short-lived symptoms and personality changes characterized by over-caution, constriction of thought, inhibition of action and impairment of the ability to experience gratification (reward)	Authors consider a double trauma, i.e., the child’s personal trauma and its traumatic effect on the caretaking parent(s). These changes will be carried forward and generalise, constraining later personality formation
Spiegel, 2000 [25]	PS.	Evaluate the effectiveness of a school-based dog bite safety primary program for elementary school children in Maryland, US	The pilot program was assessed through pre/post program questionnaires completed by 486 students	The program demonstrated ↑ effectiveness in teaching children how to prevent potentially dangerous situations with dogs. Students showed ↑ awareness of risk and appropriate behaviours	Dog bite safety programs in primary schools can be effective in promoting children’s safety. Further research is needed to monitor actual changes in children’s behaviour
Overall et al., 2001 [14]	RS.	To analyse demographics, epidemiology, injuries and risks associated with dog bites to humans	Data on dog bite cases across a wide geographic area over a 5 year period collected by hospitals, veterinary clinics, and animal control services	↑ incidence of dog bites. Children < 10 years and adults aged 20–40 years (unexpectedly) were most affected victims. Common injuries: extremities, face and neck	Significance of public education regarding safety around dogs and the importance of responsible pet management. Prevention strategies need to be refined
Anyfantakis et al., 2009 [29]	CR.	Case presentation: A 4-year-old girl admitted to the University Hospital of Crete for dog-bite related injuries developed selective mutism and acute PTSD	Clinical observation	This case highlights the need for greater awareness of the psychological effects of dog bites in children among healthcare providers and caregivers	Childhood psychological distress following dog bites is not extensively documented. This case emphasises the importance of counselling, psychological support, follow-up care and parents being aware of post-traumatic psychiatric morbidity in children after physical trauma
Daniels at al., 2009 [30]	CS.	To examine hospital incidence, charges, and characteristics of dog bite injuries among children by age.	An electronic hospital database used to identify patients < 18 years from 1999–2006	1347 children < 18 were treated for dog bites. Most were treated and discharged from the ED	Injuries to the head/neck region ↑ the odds of requiring 23-h observation and age < 5 years ↑ the odds of being admitted as an inpatient
Meints & De Keuster, 2009 [31]	ES.	To evaluate the effectiveness of the ‘Blue Dog’ CD in teaching dog-bite prevention to children < 7 years	96 children aged 3–6 used ‘Blue Dog’ CD in 3 phases with half receiving verbal feedback and the others practice with parents; re-test after 2 weeks	All age groups demonstrated ↑ safe choices post-training. Children < 6 years benefited from parental involvement, while verbal feedback did not impact effectiveness	Children 3–6 learned safety messages from the “Test Yourself” module on the Blue Dog CD with older children performing better
Shields et al., 2009 [32]	RS.	To analyse dog bite-related fatalities in Kentucky, focusing on the role of forensic odontology in investigating these incidents	11 cases of dog bite-related fatalities in Kentucky (1991–2005). Postmortem examinations in 8 cases; forensic odontology in 4 cases	Most fatalities involved multiple blunt and sharp force injuries (primarily head/neck). Common breed: Pit bulls. Common victims: children < 6 years	The study emphasizes the importance of responsible dog ownership and prevention strategies to reduce dog bite-related fatalities
Reisner et al., 2011 [10]	CS.	To characterise the behavioural circumstances of dog bites	Interview children ≤ 17 years presenting with dog bite injuries to The Children’s Hospital of Philadelphia (Penn., US)	Older bitten children were unfamiliar with the dog and not interacting with them. Face bites predominated (70%) in the younger group (<7 years), bites to extremities predominated (72%) in the older group	Recognition of the two distinctive behavioural and circumstantial subgroups of dog bites that emerged can lead to more effective prevention strategies
Dixon et al., 2012 [33]	CS.	To assess children’s knowledge about preventing dog bites and to understand parental desires for dog bite prevention education	Participants: 5- to 15-year-olds and their parents or guardians who visited a paediatric ED for non-urgent complaints or dog bites	43% of children failed the knowledge test. Children and those with white parents had ↑ odds of passing the test. >70% of children did not receive dog bite prevention education; 88% of parents wished receiving education	This study highlights poor knowledge about dog bite prevention, especially among younger children and among those with non-Caucasian parents
Fein et al., 2018 [9]	CS, RS.	This study aims to identify risk factors for paediatric dog bites using NTDB. Hypotheses: younger boys at ↑risk, with most bites occurring at home and resulting in moderate to severe injuries (particularly face/neck)	7.912 children, aged 0–17 years with ICD-9. Datasets from 2007 to 2014 were used and included patient’s gender, age, ICD-9 primary and location E-codes, AIS body region and AIS severity	Age of the child predicted where the incident occurred, the severity of the injury and the body region affected, all with statistical significance. Additionally, the body region of the injury predicted its severity	This study’s population risk factors profile suggests targeting educational initiatives on responsible dog ownership towards parents of young children as a preventive measure
Caffrey et al., 2019 [11]	CS.	To analyse factors contributing to dog bite severity using data from Calgary; Alberta, Canada	Data from 2012–2017 on dog aggression accidents were obtained using the Chameleon customisable software. The Dunbar Scale applied to classify accident severity	2012–2017: 2723 accidents of chases or bites involving humans; severity: 51% low; 35% medium; 13.5% high. Accidents occurred on owner’s property, in off-leash parks or at home. Male older adults ↑ odds of moderate to severe bite	Severe accidents are common at home and affect all age groups (children, youths, adults). Need for targeted education for both dog owners and the public to prevent bites, through supervision of children around dog and programs for older adults
Parente et al., 2021 [34]	RS.	To evaluate whether stress upon domestic dogs resulted in an increase of dog bites in the paediatric population	From records of patients admitted to paediatric EDs for dog bites 2014–2020, total mean dog bites for the years 2014–2019 and mean number per single month with 2020 data were compared	From 2014–2019, 32% were from familiar dogs and 68% from foreign dogs. In 2020, there was a significant ↓ in bites from foreign dogs and an ↑ in bites from familiar dogs compared to the average for the previous period. ↑ anxiety and depression in both children, families and dogs due to the lockdown were put in relation to the ↑ incidence of dog bites in 2020	The number of family dog bites in children ↑ in 2020, especially during the lockdown period. Paediatricians had the task to educate parents during the pandemic on the importance of continuous child supervision during their interactions with dogs. Dog owners should manage their dog’s increasing anxiety

Abbreviations: AIS Abbreviated Injury Scale; CR, case report; CS, cross-sectional study; ED, Emergency Department; ES, Experimental Study; ICD-9, International Classification of Diseases 9th edition; NTDB, National Trauma Data Bank; PS, Pilot Study; PTSD, post-traumatic stress disorder; RS, retrospective study; US, United States of America; ↓, decreased, low; ↑, increased, high.
